# Cannabis, Cannabinoids, and Stroke: Increased Risk or Potential for Protection—A Narrative Review

**DOI:** 10.3390/cimb46040196

**Published:** 2024-04-04

**Authors:** Caroline Carter, Lindsay Laviolette, Bashir Bietar, Juan Zhou, Christian Lehmann

**Affiliations:** Department of Anesthesia, Dalhousie University, Halifax, NS B3H 4R2, Canada; caroline.carter@dal.ca (C.C.); lindsay.laviolette@dal.ca (L.L.); bashir.bietar@dal.ca (B.B.); juan.zhou@dal.ca (J.Z.)

**Keywords:** cannabis, cannabinoid, stroke, neuroprotection

## Abstract

Worldwide, approximately 15 million people per year suffer from stroke. With about 5 million deaths, stroke is the second most common cause of death and a major cause of long-term disability. It is estimated that about 25% of people older than 85 years will develop stroke. *Cannabis sativa* and derived cannabinoids have been used for recreational and medical purposes for many centuries. However, due to the legal status in the past, research faced restrictions, and cannabis use was stigmatized for potential negative impacts on health. With the changes in legal status in many countries of the world, cannabis and cannabis-derived substances such as cannabinoids and terpenes have gained more interest in medical research. Several medical effects of cannabis have been scientifically proven, and potential risks identified. In the context of stroke, the role of cannabis is controversial. The negative impact of cannabis use on stroke has been reported through case reports and population-based studies. However, potential beneficial effects of specific cannabinoids are described in animal studies under certain conditions. In this narrative review, the existing body of evidence regarding the negative and positive impacts of cannabis use prior to stroke will be critically appraised.

## 1. Introduction

Globally, stroke is a major cause of death and disability. Therefore, understanding factors that contribute or mitigate CNS injury-related pathologies remains paramount in reducing the disease burden. Cannabinoids from Cannabis sativa (i.e., phytocannabinoids) are the most widely used psychoactive drugs today [1]. About 180 million people consume cannabis annually and this number has been steadily increasing as this substance has become legalized in different places around the world [2]. There is a large public interest in potential therapeutic effects of cannabinoids [3]. Currently, over 100 cannabinoids have been isolated [4]. Tetrahydrocannabinol (THC) and cannabidiol (CBD) are the most common, and best studied, phytocannabinoids [5]. THC is considered the main psychotropic component of the cannabis plant [6] and its effects include cognitive impairment, altered sense of time, or mood changes [7]. CBD is nonpsychotropic, though its mechanisms are much less studied [7]. Therapeutic actions of THC and CBD include the ability to act as anti-inflammatory agents, and neuroprotection [8]. Adverse effects of cannabis include impact on blood pressure, memory, psychomotor performance, and psychosis, though acute toxicity of cannabis is low [9].

Cannabinoids can be divided into endocannabinoids, synthetic, and phytocannabinoids [7]. Synthetic cannabinoids include prescription and illicit compounds. Spice and K2 are two synthetic cannabinoids with a high potential for abuse, and they exhibit high affinity for the CB1 receptor [7].

Endocannabinoids are the endogenous counterparts of phytocannabinoids [4] with the two G protein-coupled receptors, CB1 and CB2, as the endogenous cannabinoid receptors [10]. The CB1 receptor is found mostly throughout the nervous system, and the CB2 receptor is mostly found in the immune system [6]. Both CB1 and CB2 receptor activation causes inhibition of adenylate cyclase activation by Gi or Go signaling processes, which results in an overall reduction in cyclic adenosine monophosphate (cAMP) production [6]. In the CNS, activation of CB1 is associated with a reduction in neurotransmitter (NT) release at central synapses via a retrograde signaling mechanism involving inhibition of presynaptic voltage-dependent Ca channels. Both pre- and postsynaptic neuronal CB1 activation have been demonstrated to be neuroprotective in various neurodegenerative CNS disorders and may involve, in part, a reduction in excitotoxic NT release, modification in the glial release of proinflammatory mediators, and improved blood flow to the damaged brain. CB2 are expressed on innate and adaptive immune cells and other non-neuronal cells, including CNS resident microglial cells, glia, and endothelial cells. In contrast to CB1, and in keeping with the restricted expression of this receptor to primarily non-neuronal cells, activation of CB2 is nonpsychoactive.

Two commonly studied endogenous cannabinoids that bind to these receptors include N-arachidonoylethanolamide (AEA), and 2-Arachidonoylglycerol (2-AG) [11]. AEA and 2-AG are lipids that are not stored in vesicles prior to their release [7]. AEA is produced in response to certain stimuli from glycerophospholipids by N-acyltransferase or N-acyl-phosphatidylethannolamine-specific phospholipase D (NAPE-PLD) [11]. AEA is degraded by hydrolysis to free fatty acids and ethanolamine by fatty acid amide hydrolase (FAAH) [11]. 2-AG is formed from arachidonic acid containing membrane phospholipids by three different pathways, diacylglycerol (DAG) by the enzyme DAG lipase, lipoprotein A (LPA) via LPA phosphatase, or lysophosphatidylinositol (LPI) via lysophospholipase C [11]. 2-AG is degraded to arachidonic acid and glycerol by hydrolysis catalyzed by different enzymes, including FAAH and monoacylglycerol lipase (MAGL) [11]. Phytocannabinoids, including THC and CBD, also elicit their effects by binding to the cell membrane receptors of CB1 or CB2 [12]. THC has a lack of specificity for the CB1/CB2 receptor [13] and activates both receptors. THC also has the highest potency at these receptors, compared to any other identified phytocannabinoids [14]. The endocannabinoid system plays many roles in health and diseases, including neurological disorders such as stroke.

A stroke occurs when the blood supply to the brain has been disturbed due to ischemia or hemorrhage [13], which can lead to neurological deficits or death [15]. An ischemic stroke is much more common than hemorrhagic stroke, which is responsible for less than 20% of all strokes [16]. There are many different factors that can lead or contribute to the onset of a stroke, including drugs of abuse [17]. Worldwide, approximately 15 million people per year have a stroke. With about 5 million deaths, stroke is the second most common cause of death and a major cause of long-term disability [18]. It is estimated that about 25% of people older than 85 years will develop stroke.

Many countries permit cannabis use for both recreational and medicinal use [19]. However, there is also significant research reporting negative impacts that cannabis can have under certain conditions [20]. In the context of stroke, cannabis use has the potential to impact incidence and outcome of the condition. As some cannabinoids can provide neuroprotection, there has been indication, mostly through animal studies, that prestroke cannabinoids can have positive effects such as reduced infarct size [21]. Other evidence, mostly collected from case reports or population-based studies, suggests that cannabis use could be a risk factor for stroke and worsening patients’ outcomes [7]. This narrative review will contrast evidence to support both opposing views.

## 2. Negative Impacts of Cannabis Use on Stroke

Several studies have claimed a link between cannabis use and the risk of having a stroke. The incidence of cannabis-related strokes has been increasing in recent years, which coincides with cannabis being more readily accessible and widely used [22,23].

### 2.1. Epidemiology

There is no consistent definition of a cannabis user in the literature. In general, a cannabis user is defined as someone who had reported smoking cannabis recently before data collection for the studies examined. When referring to a chronic user, this entails someone who had smoked cannabis before their stroke as well as regularly throughout their life. When looking at the stroke incidence in cannabis users, many reports indicate an increased risk of stroke for people who use cannabis regularly [23,24,25,26,27]. The increase in cannabis users was reported as 1.8 [26], 2.3 [23], and most commonly 4.5 to 5-fold [10,27] in comparison to nonusers [7,24,25,26] throughout different studies [7,26]. In addition, the severity of stroke was pronounced in cannabis users, evidenced by significantly more hospitalizations in the cannabis users compared to nonusers [28,29].

### 2.2. Age Groups

Most of the data collected thus far point to a link between young cannabis users and an increased risk of stroke [29], with the biggest at-risk age group between 25–34 years of age [30]. In a study looking at case reports of young people (mean age: 32 years) who had a stroke, 81% reported cannabis use [25,31]. In another study of 23 case reports of cannabis-induced stroke, the mean age was 28 [32]. Cannabis use in stroke has been found to be much more prevalent in the younger population [1], and cannabis use can be considered as an independent risk factor in stroke for ages 18–55 [33,34]. Among the cannabis related strokes, 84% of the youth were found to have particularly more complications [20]. Using the national inpatient sample dataset (2007–2014), Desai et al. identified trends amongst cannabis users and found that there was a 13.92% relative increase in stroke amongst young cannabis users (18–49 years) as compared to nonusers. This effect was most noticeable in males and was not significant in females [28]. In summary, young males that use cannabis are more likely to be hospitalized for stroke.

### 2.3. Dose, Frequency and Time Dependency

Swetlik et al. reported that among individuals who use cannabis, the occurrence of ischemic stroke was 1.2%, and hemorrhagic stroke was 0.3%, which is higher than the rates observed in people who do not use cannabis (where the prevalence was 0.8% for ischemic stroke and 0.2% for hemorrhagic stroke) and stated that there is insufficient information regarding a dose responsive relationship [35]. The negative impact of cannabis use on stroke appears to be frequency- and time-dependent [13]. In a study of case reports, 81% of the cases exhibited a temporal relationship between the time that the patient had last smoked and the occurrence of stroke [22]. Studies report that the hour immediately after smoking is a critical time point [36]; the risk of stroke increases significantly by about 4.8-fold [26]. When compared with a group of nonusers, people who use and had just smoked cannabis had an up to 5 times increase in risk of a stroke [36]. Evidence also suggests that the incidence of stroke is related to the amount of cannabis used, with heavy use having the most frequency of stroke [37]. Recent and heavy cannabis use has been found to be the most linked with stroke [38,39,40]. Infrequent use of cannabis also does not appear to influence the stroke risk, compared to nonusers and heavy users [41]. One report describes a 4.7-fold increased risk of stroke in patients who use cannabis weekly or more then weekly [42]. In a study looking at people’s marijuana consumption after having a stroke, it was reported that in one quarter of the patients, their use had increased significantly in the days leading up to stroke [43]. Stroke tends to occur most often in frequent and heavy users [44,45], and there is lots of evidence to suggest a dose-dependent or temporal relationship in stroke. This may indicate, as well, that small doses may have little or no effect in increasing the risk of a stroke. In summary, while some studies have reported on the effect of frequency and temporality of cannabis use on the risk of stroke, there are few reports on the dose ingested/inhaled and the impact that has on stroke risk. This is due to the difficulty of quantifying the ingested dose at the time of hospital admission.

### 2.4. Co-Consumption

Though many studies have reported a link between cannabis use and the occurrence of stroke, the impact of other substance (co-)abuse needs to be considered as well, since many cannabis users also used cocaine, amphetamine, or other psychostimulant drugs, as well as alcohol and tobacco, and some strokes occur with multidrug use [13,22]. For example, stimulants such as amphetamines and cocaine, as well as their derivatives, were associated with ischemic and hemorrhagic stroke with different mechanisms [22]. In a study of cannabis use in pregnant women, cannabis use during pregnancy was associated with a greater risk of ischemic stroke, whereas cannabis use before pregnancy was associated with hemorrhagic stroke, compared with women not using cannabis [46].

When alcohol and tobacco covariates were adjusted for, the association between cannabis use and stroke was no longer present [1,47]. Looking at young people who had had a stroke, 84% of the cases showed a link between cannabis use and stroke, without accounting for other substances [20], making it unknown whether cannabis use and stroke exhibits this strong association, or if it is better accounted for with other substances. In addition, cannabis users more likely had underlining health conditions or had increased risk factors including hypertension, obesity, coronary artery disease, and heart failure [33]. It was reported that cannabis use independently predicted the risk of heart failure in patients aged 18–55 years old, although the clinical association between cannabis use and development of heart failure is likely multifactorial.

### 2.5. Mechanisms of Action

Different mechanisms of action have been hypothesized to elucidate the link between cannabis and stroke (see Table 1). However, there is currently no proven mechanism to fully explain this link [17]. Most of the mechanisms assume that cannabinoids are altering cerebral perfusion through impact on blood pressure or clot formation [48]. Cannabis-related blood pressure changes include mechanisms such as vasoconstriction or vasospasm, as well as alterations of arterial blood flow through vasodilation [1,7,13,16,27].

The most evidence for potential mechanisms of cannabis-induced stroke exists for the reversible cerebral vasoconstriction syndrome [1,2,5,7,11,13,16,21,27,49,50]. Reversible cerebral vasoconstriction syndrome is a term that encompasses many syndromes related to vasoconstriction, such as vasospasms, that can later lead to the occurrence of ischemic stroke [51]. It has been found that reversible cerebral vasoconstriction syndrome was reversed when cannabis use was ceased [52]. In a prospective study of 48 young patients who had a stroke, reversible cerebral vasoconstriction was found to be a mechanism, and in follow-up, this effect of vasoconstriction was reversed in those who had stopped their cannabis use [27]. The cerebrovascular effect of cannabis was found to be correlated with an increased pulsatility index and systolic velocities, both linked with cerebral vasoconstriction [49]. Increasingly, oxidative stress, endothelial-damage-associated hemodynamic dysfunction, procoagulant effects, and mitochondrial dysfunction have been reported to be contributors to cannabis-induced angiopathy. THC, in particular, has been implicated in causing much of brain mitochondrial respiratory chain dysfunction and increases in oxidative stress [7,50,53]. THC has been hypothesized to influence coagulation leading to clot formation and subsequent stroke [30]. This is also related to other mechanisms of cannabis-induced stroke, including atherosclerosis and dysfunctional platelet aggregation [1,13,16]. In animal studies to assess the mechanisms of THC, it was found that the cerebral blood flow was reduced after administration of THC, though this study did not assess if this later caused strokes in the animals [54]. THC has been found to cause hypotension as well as vasospasm and cerebral infarction [55]. Some studies looking at THC causing hypotension found that it can lead to compensatory vasoconstriction and reversible cerebral vasoconstriction syndrome [13,56].

Another proposed mechanism of cannabis-induced stroke is activated platelet aggregation, which may contribute to the formation of a cerebral blood clot [57]. Stroke patients with high-dose cannabinoid consumption showed increased platelet aggregation, and platelets were found to be positive for both the CB1 and CB2 receptor, indicating that platelets may represent a way that cannabis is inducing stroke [58].

Atherosclerosis and atherosclerotic plaques are other potential sources for clot-formation-inducing ischemic stroke related to cannabis use. This mechanism has caused some controversy in the literature. Studies have found that the CB1 receptor activation may contribute to plaque development in blood vessels, while the CB2 receptor contributes to reduced plaque development, providing a protective effect [59]. Further research is needed to understand the exact mechanism and effects of plaque development related to the cannabinoid receptors.

**Figure 1:**
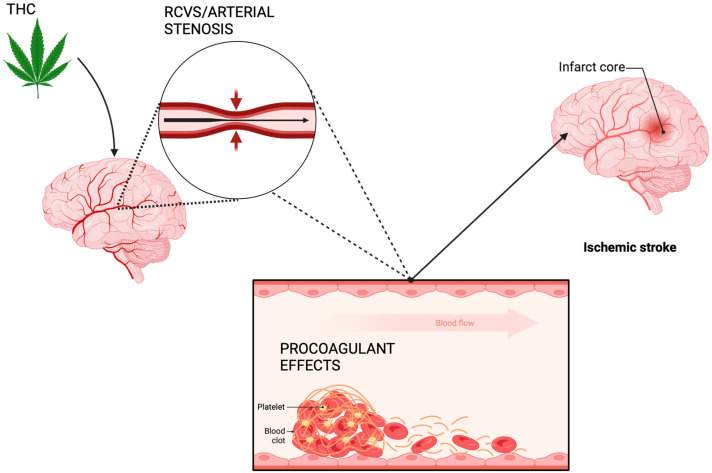
Mechanisms of cannabis-induced angiopathy leading to ischemia.

Intracranial arterial stenosis is frequently discussed in the literature in the context of cannabis-induced stroke. This mechanism works on the premise that THC is causing thickening of the arterial vessel walls, leading to narrowing of the vessel lumen and reduced blood flow to the brain, which could contribute to causing an ischemic stroke [60]. Interestingly, some studies have found a link between arterial stenosis and young cannabis users who have had a stroke [61]. In a study examining cannabis users compared to nonusers, arterial stenosis was found to be a statistically significant risk factor for stroke; however, this link only existed among the younger population [62]. This mechanism could be a promising area for future research because it is establishing a link between the apparent most at-risk age group of young adults and arterial stenosis being observed in a significant amount of the cases of stroke. Figure 1 summarizes the main contributors to cannabis-induced angiopathy.

However, since vasoconstriction does not equal complete vascular occlusion, as in ischemic stroke, further research needs to be carried out to establish the link between the mechanism of reversible vasoconstriction and its contribution to stroke to support this hypothesized method.

## 3. Potential Positive Effects of Cannabinoids on Stroke Outcome

There is growing body of evidence suggesting that the endogenous cannabinoid system can provide an array of potential benefits, particularly in the role of neuropro-tection for stroke. The majority of the in vivo results supporting these positive effects have been found in preclinical research. There have been limited studies involving human subjects performed within this field [4].

### 3.1. CB1 Receptor Agonism

Studies have shown that CB1 receptor activation can reduce neuronal damage. One of the studies examined the effect of the unspecific cannabinoid receptor agonist, WIN 55,212-2, on neuronal protection using a rat ischemic reperfusion model [63]. The ischemic injury was generated by occlusion of the common carotid artery (CCA) followed by reperfusion. The animals who received pretreatment of the agonist had increased neuronal survival compared with rats who did not receive WIN55,212-2, particularly within the stratum radiatum CA1 region of the hippocampus. Coadministration of the CB1 antagonist, SR141716A, reversed this neuroprotection, suggesting that the neuroprotection is linked to the CB1 receptor. Within the same study, researchers also observed CB1-related effects in the middle cerebral artery (MCA) occlusion model. Pretreatment with WIN55,212-2 showed an approximate 30% decrease in infarct size [63].

In another study, using electroencephalography (EEG) and spontaneous motor activity scores, gerbils treated with CP-55940, a CB1 agonist, before carotid artery occlusion showed a protective effect against EEG flattening. This effect was not present when a CB1 antagonist (SR141716A) was given, indicating direct CB1 involvement [64].

The neuroprotective potential of the CB1 receptor has also been supported by research utilizing cannabinoid receptor knockout mice. Following MCA occlusion, the mice that did not possess the CB1 receptors experienced increased mortality and more severe neurological damage. The knock-out mice displayed threefold larger cerebral infarcts and an increased number of behavioral deficits. This finding supports an endogenous neuroprotective role of the CB1 receptor [65].

**Figure 2:**
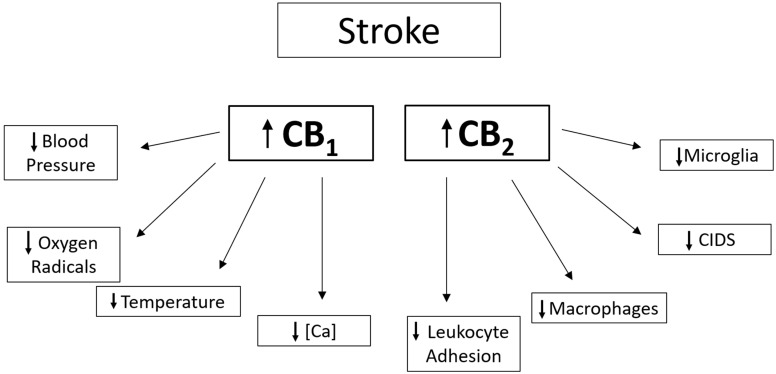
Mechanisms of action for neuroprotection by CB1 and/or CB2 receptor activation. Information from [49,63,67,68,69].

There have been several hypotheses about the exact mechanisms of cannabinoid-related neuroprotection (see Figure 2). CB1 receptors are linked to several signaling pathways, including the inhibition of calcium channels via G-protein coupled receptors. These channels are involved in the release of the neurotransmitter, glutamate, which has been shown to be related to neuronal death in several hypoxic and ischemic models [63]. Research involving rat hippocampal cultures has demonstrated that activating CB1 can inhibit the release of glutamate presynaptically, thus protecting against glutamate-induced excitotoxicity [66].

### 3.2. CB1 Receptor Antagonism

There remains uncertainty on the specific role of the CB1 receptor within ischemic stroke models due to evidence of protective effects utilizing CB1 receptor antagonists. Hansen et al. (2002) [67] demonstrated that a CB1 receptor blockade reduced infarct area and the number of degenerating neurons in a neonatal NDMA-induced damage model. The mice that were given the SR141716A, a CB1 receptor antagonist, prior to NDMA exposure, showed reduced damage within the cerebral cortex as well as the thalamus. The protective effect of the antagonist was reversed by co-administering a cannabinoid receptor agonist [67]. A similar finding was produced when the SR141716 antagonist was given to rats before MCA occlusion. The rats that received the antagonists displayed a significant reduction in cerebral infarct volume. Another experimental group was administered with WIN55,212-2 and the brain damage was not affected, showing no change compared to vehicle [68]. Reichenbach et al. (2016) [69] utilized the SR142716 antagonist in the photothrombotic model of cerebral ischemia. The antagonist was administered prior to the induction of injury. The data demonstrated a reduction in infarct volume as well as a decrease in neurological impairment scores when compared to controls [69].

Knowles et al. administered the CB1 receptor antagonist, AM251, to rats prior to occlusion. This specific antagonist has been found to block endocannabinoid function and produce no agonistic effects. Researchers examined neuronal damage and hormone expression. They found that pretreatment with AM251 lessened CA1 injury and behavioural changes, as well as reduced ischemic impacts on dopamine receptor expression and corticotropin-releasing hormone [70].

One of the possible mechanisms of the neuroprotective effects of CB1 receptor blockade is related to the receptor’s role in the release of the neurotransmitter GABA. CB1 receptors have high expression on GABA neurons and these receptors’ activity inhibits neurotransmitter release [71]. It has been found that upregulating GABA signaling aids in reducing injury in ischemic rat models; thus, blocking CB1 receptors should aid in producing this effect [72].

The different findings of CB1 agonism versus antagonism regarding neuroprotective effects are contradictory. There are several potential explanations, including differences in experimental methodology. For example, various anesthetic compounds are utilized for the experiments; route of drug administration may also play a role, e.g., intraperitoneal versus an intravenous injection. Differences in animal species and the type of ischemic model utilized should also be considered. Another important factor is the possibility that the agonists or antagonists may be acting on receptors that are outside of the endocannabinoid system (off-target effects).

### 3.3. CB2 Receptor Agonism

Growing evidence suggests that CB2 receptor activation provides neuroprotective effects. The CB2 agonists, O-1966 and O-3853, given before MCA occlusion have been shown to significantly reduce infarct size in mice. Motor function scores, taken 24 h after ischemia, were also improved in the treated mice when compared to the untreated control group [73]. These results are specific to CB2 as the agonists utilized have a low affinity for CB1 receptors. The study also examined the role of the CB2 agonists on leukocyte–endothelial interactions. CB2 activation was found to be related with decreased rolling and adhesion to vascular endothelial cells [73]. This conclusion has been supported by investigation of the immunomodulatory role of CB2 receptors in central nerve system injury. Sultana et al. found that in a CNS injury model, when the CB2 agonist HU308 was administered, the brain injury size was reduced, and leukocyte response was also restored compared to a control group with no treatment [74]. Ronca et al. also utilized the CB2 agonist O-1966 which was injected prior to the photoinjury model. Researchers also investigated injection at time points post ischemia. In all groups, they found a smaller infarct volume and protection against cognitive deficits [75]. Yu et al. utilized an MCA occlusion stroke model in rats and administered pretreatment with the CB2 agonist, AM1241. The agonist was administered prior to occlusion, and brain infarction along with neurological scores were assessed. AM1241 was found to reduce infarct size and deficits. Interestingly, the authors also assessed whether the agonist would produce the same effect if administered post occlusion. They found no behavioral improvement or reduction in infarct when the agonist was given 2–5 days following occlusion, indicating a time-dependent neuroprotection. These findings can be primarily attributed to CB2 as the AM1241 agonist has a 100-fold selectivity over the CB1 receptor [35].

### 3.4. CB2 Receptor Antagonism

Post stroke, the immune system mounts an inflammatory response. To limit neuroinflammation, depression of exaggerated immune response is initialized in parallel. This well-balanced process can be dysregulated, leading to systemic suppression of the peripheral immune response and increased susceptibility to infection, known as CNS injury-induced immunodepression syndrome (CIDS) [76]. The CB2 receptor has been found to be involved in this regulatory immune response [74]. Using a cerebral hypoxia ischemia (HI) model, the effect of CB2 receptor inhibition with AM630 on CNS injury-induced immunodeficiency syndrome (CIDS) was studied. Leukocyte activation was measured in different groups following endotoxemia challenge with and without AM630 treatment [77]. This study found that mice with endotoxemia challenge who had undergone HI had reduced leukocyte activation compared to the control group. The group that had undergone HI with endotoxemia along with AM630 treatment had restored leukocyte activation, indicating that blocking of the CB2 receptor could be a potential treatment for post stroke CIDS [77]. Importantly, AM630 did not lead to an increased infarct size.

In contrast, in a study looking at the effects of WIN55,212-2 in hypoxia ischemia of rats, it was found that WIN55,212-2 had a protective role, and administration of the CB2 antagonist SR144528 reversed the neuroprotective effects provided by WIN55,212-2 indicating that CB2 was necessary for this provided protection [78]. The CB2 receptor has been found to improve CIDS by reducing the initial inflammatory response, which in turn lessens the counter regulation and immunosuppression. In a study using HU308, a CB2 agonist, as a pretreatment in a stroke model, the local inflammatory response was reduced, and in turn, CIDS was attenuated [74]. This same study also tested the effects of late administration of AM630 post stroke and showed that delayed CB2 inhibition leads to improvement of post-stroke outcomes [74]. Therefore, in the case of CIDS, it is likely time-dependent on whether CB2 activation can is beneficial or detrimental.

### 3.5. Co-Antagonism of CB1 and CB2

The effects of different CB1 and CB2 antagonism or agonism are summarized below in Table 2.

Ward et al. hypothesized that knocking out both the CB1 and CB2 receptors would increase infarct size and they performed this experiment utilizing the MCAO model in male mice. Surprisingly, they demonstrated that these mice possessed a reduced infarct size and improved recovery. Researchers suggest that in order to compensate for this loss (of the cannabinoid receptors) and maintain homeostasis, there are changes within other pathways, such as the eicosanoid system [79].

### 3.6. Limitations and Conclusions

As there were limited data on the topic of cannabis use and stroke, a systematic or scoping review was not able to be completed, and, thus, a narrative review was carried out.

Most findings related to negative side effects or outcomes of cannabis use in stroke were retrieved from case reports, specifically from hospitalization records. These case studies are often broad with different definitions of usage, unclear methods of cannabis consumption, or no specific information on the cannabis strain that was utilized. Differences in the strain, cannabinoid compounds, and method of consumption involved have the potential to yield varying effects from usage. Terms such as “users” and “nonusers” are often listed without clarification on amount or frequency of usage, and dose dependency is an important factor in relation to cannabis use its link with stroke. “Chronic consumption” has also been listed in several reports without a clear operational definition of what this entails, such as how often and how much one needs to be consuming to fit the criteria of a chronic user. Co-consumption is often overlooked, as studies have mentioned that many cannabis users may also utilize other substances concurrently which may better explain the negative impact that cannabis use has appeared to show. Though many studies have mentioned that there is a significant correlation between cannabis use and stroke, this does not mean that this is necessarily related to causation. Without proper analysis of these factors, it is difficult to be certain of the negative effects of cannabis usage on stroke.

Despite the abovementioned limitations of the available data, it is prudent for healthcare providers to advise patients on the potential risks of recreational cannabis use. According to our search of the literature, a preventive effect of cannabis use for stroke has not been shown in any study. The higher risk of cannabis-related stroke in younger patients is concerning.

In contrast, there is potential for the endocannabinoid system (ECS) to act as drug target for patients who had a stroke. However, more studies are needed to elucidate the exact role of the ECS in stroke. In particular, the long-term effects of ECS-directed treatment are not known. In particular, the impact of CB2 modulation on systemic immune response is a potential area of concern. Without those mechanistic studies, clinical studies are at high risk of failure and detrimental outcomes.

The legalization of cannabis for recreational and medical use in many jurisdictions has contributed to decriminalization and changed the (negative) stigma of cannabis. It is now the mandate of medical research to provide the scientific data for potential beneficial effects or detrimental side effects of this drug. Stroke will be an important area of research on cannabis in the future.

## Figures and Tables

**Table 1:** Potential mechanisms of action for cannabinoid-induced stroke.

Vasculature	Clotting/Thrombosis
Reversible cerebral vasoconstriction syndrome	Intracranial arterial stenosis
Vasospasm, vasoconstriction	Atherosclerosis
Arterial blood flow alterations	Platelet aggregation

**Table 2:** Summary of potentially positive effects of cannabinoids on stroke outcome.

Mechanism	Compound	Model	Outcome	Reference
CB1/2 Agonist	WIN55,212-2	CCAO & MCAO	Reduced infarct volume	[63]
CB1 Agonist	CP5590	CCAO	Protective effect against motor activity damage	[64]
CB1 Antagonist	SR141716A	NDMA	Reduced infarct volume	[67]
CB1 Antagonist	SR141716A	MCAO	Reduced infarct volume	[68]
CB1 Antagonist	SR141716A	Photothrombotic	Reduced infarct volume	[69]
CB1 Antagonist	AM251	Global Ischemia		[70]
CB2 Agonist	O-1966, O-3853	MCAO	Reduced infarct volume and improved motor function	[73]
CB2 Agonist	O-1966	Photoinjury	Reduced infarct volume and protection against cognitive deficits	[75]
CB2 Agonist	AM1241	Global Ischemia (4VO)	Reduced infarct volume and a decrease in neurological deficits	[35]

## References

[1] Falkstedt D., Wolff V., Allebeck P., Hemmingsson T., Danielsson A.-K. (2017). Cannabis, Tobacco, Alcohol Use, and the Risk of Early Stroke. Stroke.

[2] Singh A., Saluja S., Kumar A., Agrawal S., Thind M., Nanda S., Shirani J. (2018). Cardiovascular Complications of Marijuana and Related Substances: A Review. Cardiol. Ther..

[3] Maurya N., Velmurugan B.K. (2018). Therapeutic applications of cannabinoids. Chem. Interact..

[4] Latorre J.G.S., Schmidt E.B. (2015). Cannabis, Cannabinoids, and Cerebral Metabolism: Potential Applications in Stroke and Disorders of the Central Nervous System. Curr. Cardiol. Rep..

[5] Page R.L., Allen L.A., Kloner R.A., Carriker C.R., Martel C., Morris A.A., Piano M.R., Rana J.S., Saucedo J.F., On behalf of the American Heart Association Clinical Pharmacology Committee and Heart Failure and Transplantation Committee of the Council on Clinical Cardiology (2020). Medical Marijuana, Recreational Cannabis, and Cardiovascular Health: A Scientific Statement from the American Heart Association. Circulation.

[6] Cristino L., Bisogno T., Di Marzo V. (2020). Cannabinoids and the expanded endocannabinoid system in neurological disorders. Nat. Rev. Neurol..

[7] Moustafa B., Testai F.D. (2021). Cerebrovascular Complications Associated with Marijuana Use. Curr. Neurol. Neurosci. Rep..

[8] Amin M.R., Ali D.W. (2019). Pharmacology of Medical Cannabis. Recent Advances in Cannabinoid Physiology and Pathology.

[9] Thanvi B.R., Treadwell S.D. (2009). Cannabis and Stroke: Is there a link?. Postgrad. Med. J..

[10] Di Marzo V., Piscitelli F. (2015). The Endocannabinoid System and its Modulation by Phytocannabinoids. Neurotherapeutics.

[11] Zou S., Kumar U. (2018). Cannabinoid Receptors and the Endocannabinoid System: Signaling and Function in the Central Nervous System. Int. J. Mol. Sci..

[12] Basavarajappa B.S., Shivakumar M., Joshi V., Subbanna S. (2017). Endocannabinoid system in neurodegenerative disorders. J. Neurochem..

[13] Tsatsakis A., Docea A.O., Calina D., Tsarouhas K., Zamfira L.-M., Mitrut R., Sharifi-Rad J., Kovatsi L., Siokas V., Dardiotis E. (2019). A Mechanistic and Pathophysiological Approach for Stroke Associated with Drugs of Abuse. J. Clin. Med..

[14] Jones É., Vlachou S. (2020). A Critical Review of the Role of the Cannabinoid Compounds Δ^9^-Tetrahydrocannabinol (Δ^9^-THC) and Cannabidiol (CBD) and their Combination in Multiple Sclerosis Treatment. Molecules.

[15] Easton J.D., Saver J.L., Albers G.W., Alberts M.J., Chaturvedi S., Feldmann E., Hatsukami T.S., Higashida R.T., Johnston S.C., Kidwell C.S. (2009). Definition and Evaluation of Transient Ischemic Attack. Stroke.

[16] Montaño A., Hanley D.F., Hemphill J.C. (2021). Hemorrhagic Stroke. Handb. Clin. Neurol..

[17] Fonseca A.C., Ferro J.M. (2013). Drug Abuse and Stroke. Curr. Neurol. Neurosci. Rep..

[18] World Health Organization Stroke, Cerebrovascular Accident. https://www.emro.who.int/health-topics/stroke-cerebrovascular-accident/index.html.

[19] Piano M.R. (2017). Cannabis Smoking and Cardiovascular Health: It’s Complicated. Clin. Pharmacol. Ther..

[20] Archie S.R., Cucullo L. (2019). Harmful Effects of Smoking Cannabis: A Cerebrovascular and Neurological Perspective. Front. Pharmacol..

[21] Fernández-López D., Faustino J., Derugin N., Wendland M., Lizasoain I., Moro M., Vexler Z. (2012). Reduced infarct size and accumulation of microglia in rats treated with WIN 55,212-2 after neonatal stroke. Neuroscience.

[22] Hackram D. (2015). Cannabis and Stroke: Systemic appraisal of case reports. Stroke.

[23] Jouanjus E., Raymond V., Lapeyre-Mestre M., Wolff V. (2017). What is the Current Knowledge About the Cardiovascular Risk for Users of Cannabis-Based Products? A Systematic Review. Curr. Atheroscler. Rep..

[24] Westover A.N., McBride S., Haley R.W. (2007). Stroke in Young Adults Who Abuse Amphetamines or Cocaine. Arch. Gen. Psychiatry.

[25] Wolff V., Jouanjus E. (2017). Strokes are possible complications of cannabinoids use. Epilepsy Behav..

[26] Middlekauff H.R., Cooper Z.D., Strauss S.B. (2022). Drugs of Misuse: Focus on Vascular Dysfunction. Can. J. Cardiol..

[27] Wolff V., Lauer V., Rouyer O., Sellal F., Meyer N., Raul J.S., Sabourdy C., Boujan F., Jahn C., Beaujeux R. (2011). Cannabis Use, Ischemic Stroke, and Multifocal Intracranial Vasoconstriction. Stroke.

[28] Desai R., Singh S., Patel K., Goyal H., Shah M., Mansuri Z., Patel S., Mahuwala Z.K., Goldstein L.B., Qureshi A.I. (2020). Stroke in young cannabis users (18–49 years): National trends in hospitalizations and outcomes. Int. J. Stroke.

[29] Johnston L., Miech R., O’Malley P., Bachman J., Schulenberg J., Patrick M. (2019). Monitoring the Future National Survey Results on Drug Use, 1975–2018: Overview, Key Findings on Adolescent Drug Use.

[30] O’keefe E.L., Peterson T.M., Lavie C.J. (2021). Reevaluating America’s Latest Pharmaceutical Trend: The Cardiovascular Risk of Cannabis. Curr. Opin. Psychol..

[31] Wolff V., Armspach J.-P., Lauer V., Rouyer O., Bataillard M., Marescaux C., Geny B. (2013). Cannabis-related Stroke. Stroke.

[32] Dabhi N., Mastorakos P., Sokolowski J.D., Kellogg R.T., Park M.S. (2022). Effect of drug use in the treatment of acute ischemic stroke: A scoping review. Surg. Neurol. Int..

[33] Kalla A., Krishnamoorthy P.M., Gopalakrishnan A., Figueredo V.M. (2018). Cannabis use predicts risks of heart failure and cerebrovascular accidents. J. Cardiovasc. Med..

[34] Cooles P., Michaud R. (1987). Stroke after heavy cannabis smoking. Postgrad. Med. J..

[35] Yu S.-J., Reiner D., Shen H., Wu K.-J., Liu Q.-R., Wang Y. (2015). Time-Dependent Protection of CB2 Receptor Agonist in Stroke. PLoS ONE.

[36] Mittleman M.A., Lewis R.A., Maclure M., Sherwood J.B., Muller J.E. (2001). Triggering Myocardial Infarction by Marijuana. Circulation.

[37] Parekh T., Pemmasani S., Desai R. (2020). Marijuana Use Among Young Adults (18–44 Years of Age) and Risk of Stroke. Stroke.

[38] Tirkey N.K., Gupta S. (2016). Acute Antero-Inferior Wall Ischaemia with Acute Ischaemic Stroke Caused by Oral Ingestion of Cannabis in a Young Male. J. Assoc. Physicians India.

[39] Volpon L.C., Sousa C.L.M.D.M., Moreira S.K.K., Teixeira S.R., Carlotti A.P.D.C.P. (2017). Multiple Cerebral Infarcts in a Young Patient Associated with Marijuana Use. J. Addict. Med..

[40] Šimůnek L., Krajina A., Herzig R., Vališ M. (2018). Cerebral Infarction in Young Marijuana Smokers—Case Reports. Acta Medica.

[41] Hemachandra D., McKetin R., Cherbuin N., Anstey K.J. (2016). Heavy cannabis users at elevated risk of stroke: Evidence from a general population survey. Aust. N. Z. J. Public Health.

[42] Rumalla K., Reddy A.Y., Mittal M.K. (2016). Recreational marijuana use and acute ischemic stroke: A population-based analysis of hospitalized patients in the United States. J. Neurol. Sci..

[43] Yau W.Y., Chu E., Lai N. (2015). Cannabis, serotonergic drug use and stroke in a 50-year-old woman. Intern. Med. J..

[44] Geller T., Loftis L., Brink D.S. (2004). Cerebellar Infarction in Adolescent Males Associated with Acute Marijuana Use. Pediatrics.

[45] Russmann S., Winkler A., Lövblad K., Stanga Z., Bassetti C. (2002). Lethal Ischemic Stroke after Cisplatin-Based Chemotherapy for Testicular Carcinoma and Cannabis Inhalation. Eur. Neurol..

[46] Auger N., Paradis G., Low N., Ayoub A., He S., Potter B.J. (2020). Cannabis use disorder and the future risk of cardiovascular disease in parous women: A longitudinal cohort study. BMC Med..

[47] Barber P.A., Pridmore H.M., Krishnamurthy V., Roberts S., Spriggs D.A., Carter K.N., Anderson N.E. (2013). Cannabis, Ischemic Stroke, and Transient Ischemic Attack. Stroke.

[48] Mathew R.J., Wilson W.H., Humphreys D., Lowe J.V., Wiethe K.E. (1992). Middle cerebral artery velocity during upright posture after marijuana smoking. Acta Psychiatr. Scand..

[49] Swetlik C., Migdady I., Hasan L.Z., Buletko A.B., Price C.M., Cho S.-M.D. (2022). Cannabis Use and Stroke: Does a Risk Exist?. J. Addict. Med..

[50] Wolff V., Schlagowski A.-I., Rouyer O., Charles A.-L., Singh F., Auger C., Schini-Kerth V., Marescaux C., Raul J.-S., Zoll J. (2015). Tetrahydrocannabinol Induces Brain Mitochondrial Respiratory Chain Dysfunction and Increases Oxidative Stress: A Potential Mechanism Involved in Cannabis-Related Stroke. BioMed Res. Int..

[51] Mikami T., Obata R., Steinberg D.I., Skliut M., Boniece I. (2021). Marijuana-related Reversible Cerebral Vasoconstriction Syndrome. Intern. Med..

[52] Thomas G., Kloner R.A., Rezkalla S. (2014). Adverse Cardiovascular, Cerebrovascular, and Peripheral Vascular Effects of Marijuana Inhalation: What Cardiologists Need to Know. Am. J. Cardiol..

[53] Choi S.-H., Mou Y., Silva A.C. (2019). Cannabis and Cannabinoid Biology in Stroke. Stroke.

[54] Richter J.S., Quenardelle V., Rouyer O., Raul J.S., Beaujeux R., Gény B., Wolff V. (2018). A Systematic Review of the Complex Effects of Cannabinoids on Cerebral and Peripheral Circulation in Animal Models. Front. Physiol..

[55] Zachariah S.B. (1991). Stroke after heavy marijuana smoking. Stroke.

[56] Testai F.D., Gorelick P.B., Aparicio H.J., Filbey F.M., Gonzalez R., Gottesman R.F., Melis M., Piano M.R., Rubino T., Song S.Y. (2022). Use of Marijuana: Effect on Brain Health: A Scientific Statement from the American Heart Association. Stroke.

[57] Turcotte C., Chouinard F., Lefebvre J.S., Flamand N. (2015). Regulation of inflammation by cannabinoids, the endocannabinoids 2-arachidonoyl-glycerol and arachidonoyl-ethanolamide, and their metabolites. J. Leukoc. Biol..

[58] Dahdouh Z., Roule V., Lognoné T., Sabatier R., Grollier G. (2012). Cannabis and coronary thrombosis: What is the role of platelets?. Platelets.

[59] Pacher P., Steffens S., Haskó G., Schindler T.H., Kunos G. (2018). Cardiovascular effects of marijuana and synthetic cannabinoids: The good, the bad, and the ugly. Nat. Rev. Cardiol..

[60] O’keefe E.L., Dhore-Patil A., Lavie C.J. (2022). Early-Onset Cardiovascular Disease from Cocaine, Amphetamines, Alcohol, and Marijuana. Can. J. Cardiol..

[61] Wolff V., Armspach J.-P., Beaujeux R., Manisor M., Rouyer O., Lauer V., Meyer N., Marescaux C., Geny B. (2014). High Frequency of Intracranial Arterial Stenosis and Cannabis Use in Ischaemic Stroke in the Young. Cerebrovasc. Dis..

[62] Wolff V., Zinchenko I., Quenardelle V., Rouyer O., Geny B. (2015). Characteristics and Prognosis of Ischemic Stroke in Young Cannabis Users Compared with Non-Cannabis Users. J. Am. Coll. Cardiol..

[63] Nagayama T., Sinor A.D., Simon R.P., Chen J., Graham S.H., Jin K., Greenberg D.A. (1999). Cannabinoids and Neuroprotection in Global and Focal Cerebral Ischemia and in Neuronal Cultures. J. Neurosci..

[64] Braida D., Pozzi M., Sala M. (2000). CP 55,940 protects against ischemia-induced electroencephalographic flattening and hyperlocomotionin Mongolian gerbils. Neurosci. Lett..

[65] Parmentier-Batteur S., Jin K., Mao X.O., Xie L., Greenberg D.A. (2002). Increased Severity of Stroke in CB1 Cannabinoid Receptor Knock-Out Mice. J. Neurosci..

[66] Shen M., Piser T.M., Seybold V.S., Thayer S.A. (1996). Cannabinoid Receptor Agonists Inhibit Glutamatergic Synaptic Transmission in Rat Hippocampal Cultures. J. Neurosci..

[67] Hansen H.H., Azcoitia I., Pons S., Romero J., García-Segura L.M., Ramos J.A., Hansen H.S., Fernández-Ruiz J. (2002). Blockade of cannabinoid CB_1_ receptor function protects against in vivo disseminating brain damage following NMDA-induced excitotoxicity. J. Neurochem..

[68] Amantea D., Spagnuolo P., Bari M., Fezza F., Mazzei C., Tassorelli C., Morrone L.A., Corasaniti M.T., Maccarrone M., Bagetta G. (2007). Modulation of the endocannabinoid system by focal brain ischemia in the rat is involved in neuroprotection afforded by 17β-estradiol. FEBS J..

[69] Reichenbach Z.W., Li H., Ward S.J., Tuma R.F. (2016). The CB1 antagonist, SR141716A, is protective in permanent photothrombotic cerebral ischemia. Neurosci. Lett..

[70] Knowles M.D., de la Tremblaye P.B., Azogu I., Plamondon H. (2016). Endocannabinoid CB1 receptor activation upon global ischemia adversely impact recovery of reward and stress signaling molecules, neuronal survival and behavioral impulsivity. Prog. Neuro-Psychopharmacol. Biol. Psychiatry.

[71] James S.P., Bondugji D. (2022). Gamma-Aminobutyric Acid (GABA) and the Endocannabinoids: Understanding the Risks and Opportunities. Natural Drugs from Plants.

[72] Xu J., Li C., Yin X.-H., Zhang G.-Y. (2008). Additive neuroprotection of GABA A and GABA B receptor agonists in cerebral ischemic injury via PI-3K/Akt pathway inhibiting the ASK1-JNK cascade. Neuropharmacology.

[73] Zhang M., Martin B.R., Adler M.W., Razdan R.K., Jallo J.I., Tuma R.F. (2007). Cannabinoid CB_2_ Receptor Activation Decreases Cerebral Infarction in a Mouse Focal Ischemia/Reperfusion Model. J. Cereb. Blood Flow Metab..

[74] Sultana S., Burkovskiy I., Zhou J., Kelly M.M., Lehmann C. (2021). Effect of Cannabinoid 2 Receptor Modulation on the Peripheral Immune Response in Central Nervous System Injury-Induced Immunodeficiency Syndrome. Cannabis Cannabinoid Res..

[75] Ronca R.D., Myers A.M., Ganea D., Tuma R.F., Walker E.A., Ward S.J. (2015). A selective cannabinoid CB2 agonist attenuates damage and improves memory retention following stroke in mice. Life Sci..

[76] Bietar B., Zhou J., Lehmann C. (2021). Utility of intestinal intravital microscopy for the study of CNS injury-induced immunodepression syndrome (CIDS). Clin. Hemorheol. Microcirc..

[77] Burkovskiy I., Zhou J., Lehmann C. (2016). Experimental Cannabinoid 2 Receptor Inhibition in CNS Injury-Induced Immunodeficiency Syndrome. Microcirculation.

[78] Hillard C.J. (2008). Role of Cannabinoids and Endocannabinoids in Cerebral Ischemia. Curr. Pharm. Des..

[79] Ward S.J., Castelli F., Reichenbach Z.W., Tuma R.F. (2018). Surprising outcomes in cannabinoid CB1/CB2 receptor double knockout mice in two models of ischemia. Life Sci..

